# Lipegfilgrastim for primary prophylaxis of febrile neutropenia in patients treated for advanced-stage classical hodgkin lymphoma: successful outcomes from a multicenter cohort study

**DOI:** 10.1007/s00277-026-06911-7

**Published:** 2026-03-18

**Authors:** Claudia Giordano, M. Picardi, F. Esposito, A. Vincenzi, N. Pugliese, A. Lombardi, F. Trastulli, R. Secchi, M. Postorino, M. Annunziata, A. Venditti, F. Pane

**Affiliations:** 1 Department of Clinical Medicine and Surgery, Federico II University Medical School, Via Sergio Pansini, 5, Naples, 80131 Italy; 2 Department of Biomedicine and Prevention, Fondazione Policlinico Torvergata, Viale Oxford, 81, Rome, 00133 Italy; 3 Hematology Unit, Hospital “Antonio Cardarelli”, Naples, Italy

**Keywords:** Hodgkin lymphoma, Febrile neutropenia, Granulocyte colony-stimulating factor, Lipegfilgrastim

## Abstract

In patients with classical Hodgkin lymphoma (c-HL) undergoing ABVD chemotherapy for advanced disease, the optimal strategy to prevent febrile neutropenia (FN)—defined as fever ≥ 38 °C with absolute neutrophil count (ANC) < 1000/mm³—remains debated. Possible prophylaxis approaches include: *i*) secondary prophylaxis with on-demand granulocyte colony-stimulating factor (G-CSF, filgrastim), *ii*) primary prophylaxis with filgrastim, or *iii*) primary prophylaxis with long-acting G-CSF formulations such as pegylated or glyco-pegylated G-CSF (lipegfilgrastim). We conducted a multicenter retrospective cohort study from 2010 to 2024 involving 450 untreated c-HL patients (Ann Arbor stage IIB-IV) scheduled for six ABVD cycles, divided into three five-year periods, each with a different G-CSF prophylaxis strategy. From 2010 to 2014, 131 patients received on-demand filgrastim when ANC ≤ 1 × 10^9/L (*on-demand*- group); from 2015 to 2019, 152 patients systematically received filgrastim six times per cycle (*filgrastim-*group); from 2020 to 2024, 167 patients received lipegfilgrastim twice per cycle as primary prophylaxis (*lipegfilgrastim*-group). A total of 85 neutropenia episodes occurred: 52 in the *on-demand*-group, 30 in the *filgrastim*-group, and 3 in the *lipegfilgrastim*-group (*P* < 0.001); FN incidence was 24%, 14%, and 2%, respectively (*P* < 0.0001). Chemotherapy disruptions due to FN were 14%, 6%, and 1%, respectively (*P* < 0.001). Grade 3 bone pain occurred in 5% of patients and was managed with analgesics. Primary prophylaxis with lipegfilgrastim significantly reduced FN rates, hospitalizations, and chemotherapy interruptions in patients with advanced-stage c-HL treated with ABVD, demonstrating improved tolerability of chemotherapy.

## Introduction

In patients with classical Hodgkin lymphoma (c-HL) receiving the multiagent chemotherapy scheme of adriamycin, bleomycin, vinblastine and dacarbazine (ABVD), a proper strategy of prophylaxis to prevent febrile neutropenia (FN) is still a controversial matter. FN is one of the most important clinical signs of infection during chemotherapy treatment and is characterized by an absolute neutrophil count (ANC) < 1000/mm^3^ and at least one temperature measuring ≥ 38 °C [1]. The National Comprehensive Cancer Network (NCCN) and the American Society of Clinical Oncology (ASCO) advise using granulocyte colony-stimulating factors (G-CSFs), which are designed to promote neutrophil differentiation and proliferation in patients undergoing cytotoxic treatment. G-CSFs from the first cycle of myelosuppressive chemotherapy, often known as primary prophylaxis, should be routinely used when the total risk of FN is more than 20%. Secondary prophylaxis, which includes *on-demand* post-chemotherapy G-CSF delivery (when ANC is < 1000/mm^3^), is recommended if the risk is less than 20% [1]. According to NCCN guidelines, ABVD is considered a chemotherapy regimen with low to medium-risk of FN thus it’s not advised to employ prophylaxis with G-CSF for routine use, but primary prophylaxis is only appropriate for a fraction of patients with patient-related risk factors (such as older age) [2].

Real-life data show a pooled neutropenia rate of 30–40% in patients with HL (stage IIB-IV) receiving ABVD regimen [1], and febrile neutropenia of approximated 7% overall (range 5% – 9.8%), or approximately 1% per cycle of ABVD. The choice to postpone treatment or continue as planned, with or without the inclusion of G-CSF and/or a concurrent decrease in the dosages of specific chemotherapy medications, has always been at the discretion of the physician [3]. However, the decision to postpone or lower the dosage of chemotherapy in response to uncomplicated neutropenia at the time of scheduled chemotherapy could result in a lower dose intensity with the risk of jeopardizing patient outcome [4, 5].

Many studies have shown that the administration of GCSFs can lower the incidence of neutropenia and neutropenic fevers in lymphoma patients receiving anthracycline-based chemotherapy, but HL patients are either absent or underweighted in these studies [6–8] and this approach has yet to be prospectively studied by means of randomized controlled trials. Moreover, for managing cancer patients undergoing chemotherapy during the COVID-19 pandemic, the European Society for Medical Oncology (ESMO) multidisciplinary expert consensus states to expand the routine indication of G-CSF for patients with intermediate (10%−20%) risk of FN and specifically for older patients with comorbidities [9].

An important issue is also the type of G-CSF to employ: filgrastim is a non-pegylated short-acting form of G-CSF, used at the daily dose of 5 µg/kg until the end of neutropenia, according to the myelosuppressive grade of chemotherapy schedules; pegfilgrastim is a pegylated long-acting recombinant form of G-CSF with extended half-life used to decrease the incidence of infections and requiring less frequent administrations (single dose administration per chemotherapy cycle); lipegfilgrastim is a novel glyco-pegylated long-acting G-CSF that was approved by the European Medicines Agency in 2013 to prevent chemotherapy related neutropenia with a once-per-cycle administration [10]. Phase III studies on the efficacy and safety of lipegfilgrastim versus pegfilgrastim and recent meta-analysis in patients receiving doxorubicin chemotherapy demonstrated a higher efficacy in terms of anti-neutropenic activity and shorter ANC recovery time for lipegfilgrastim with fewer toxicity events [11, 12].

For the lack of data in literature potentially guiding a prophylaxis strategy, we designed a multi-center retrospective cohort study to evaluate the incidence rate of FN, as the primary endpoint, analyzing three groups of patients in three 5-years consecutive periods differing on the type of prophylaxis. Secondary endpoints were FN-related chemotherapy disruption (regarding dose-dense and/or dose-intensity of the ABVD schedule), and days of hospitalization due to FN and G-CSF related side effects (grade ≥ 3 CTCAE v.5.0 toxicity criteria) in each group.

## Material and methods

### Study design

This was a retrospective study using the medical records or local database of the Hematology Unit of the Federico II University of Naples (Italy), Hematology Unit of the Torvergata hospital of Rome (Italy), and the Hematology Unit of Antonio Cardarelli Hospital of national importance of Naples (Italy). All necessary approvals were obtained from the Ethic Committee of the coordinator center Federico II University of Naples (approval protocol number: 65/2024). The acquisition of a written informed consent was obtained from each patient undergoing chemotherapy treatment. The form contained detailed information regarding the risk of the treatment.

From January 2010 to December 2024, consecutive untreated c-HL patients with Ann Arbor stage IIB-IV with clinical indication to receive 6 cycles of ABVD regimen were enrolled. Patients were stratified in three consecutive 5-years periods, in each of whom a specific prophylaxis against FN was employed.

### Real-life patients’ stratification and prophylaxis strategies

Patients’ stratification according to the different prophylaxis strategies was as follows: (1) from January 2010 to December 2014, patients undergoing secondary prophylaxis were allocated in the *on-demand*-group where G-CSF (filgrastim) was injected subcutaneously (*s.c.*) on demand, that is when ANC was ≤ 1 × 10^9^/L at the weekly cell blood count (CBC) monitoring performed from the first course of ABVD until the last course; (2) from January 2015 to December 2019, patients received primary prophylaxis with G-CSF (filgrastim) injected *s.c.* with an 6-times administration (days 3, 5, 7, 17, 19 and 21) of each 4-week cycle of ABVD from the first course of chemotherapy until the last course (*filgrastim*-group); and (3) from January 2020 to December 2024, patients underwent primary prophylaxis with lipegfilgrastim (*lipegfilgrastim*-group) administered *s.c.* as single dose on days 3 and 18 of each 4-week cycle of ABVD from the first course of chemotherapy until the last course.

### Inclusion and exclusion criteria

Inclusion criteria were: *i*) age older than 18 years; *ii*) histological confirmation of CD30 + subtype of c-HL at diagnosis; *iii*) Ann Arbor stage IIB-IV; *iv*) frontline chemotherapy treatment with ABVD regimen at standard dose (at least 1 cycle administered) for 6 scheduled cycles; and *v*) European Cooperative Oncology Group performance status (ECOG-PS) 0–2 (or 3, if due to illness).

The exclusion criteria were patients receiving a chemotherapy regimen other than ABVD, a histological diagnosis other than c-HL, a previous neoplastic disease (for which preexisting anticancer therapy was employed).

### Data collection

Data collection forms were distributed to the three centers’ data managers to record patient demographics, medical history, histological diagnosis, ECOG PS, baseline and end-of-treatment fluoro-desossy-glucose positron emission computed tomography (FDG-PET/CT), bone marrow results, chemotherapy details (regimen, dose reductions, delays), prophylactic and supportive treatments, and baseline blood tests. Collected data included blood counts, biochemistry, immunoglobulin levels, FN events, grade 3–5 and opportunistic infections, and adverse events affecting treatment delivery, G-CSF–related side effects, and chemotherapy-related deaths. Information on the duration of severe neutropenia, ANC nadir per cycle, and time to nadir and recovery (ANC ≥ 2.0 × 10⁹/L) was also retrieved.

### Study endpoints

The primary endpoint was FN incidence during each G-CSF-prophylaxis period, secondary endpoints were FN-related chemotherapy disruption (regarding dose-dense and/or dose-intensity of the schedule), days of hospitalization due to FN, and prophylaxis-related side effects (grade ≥ 3 Common Terminology Criteria for Adverse Events version 5.0 [CTCAE]).

As per the secondary endpoint of FN-related chemotherapy disruption, if the leukocyte count was less than 2.0 × 10^9^/L before a scheduled cycle, treatment cycle was delayed for at least 1 week thus a time-disruption was recorded; while, for dose reduction of at least one drug of ABVD scheme when leukocyte count was less than 1000/mm^3^ on two consecutive days between cycles, a dose-disruption was recorded.

In addition, in patients aged ≥ 60 years bleomycin dose was a priori reduced by 50% from the first cycle in order to minimize the risk of pulmonary toxicity, according to institutional practice.

### Infection diagnostic work-up

During the study periods, according to institutional post-chemotherapy supportive care guidelines, patients with FN (ANC < 1.0 × 10⁹/L and fever ≥ 38 °C) underwent a standard infection work-up. This included blood cultures (at fever onset and 24–48 h later), urine analysis, liver and renal tests, nasopharyngeal swab, and sputum culture. If fever persisted beyond 72 h, the work-up was expanded to include serum CMV-DNA, galactomannan, and β-D-glucan assays; chest X-ray or CT scan with bronchoscopy and bronchoalveolar lavage when indicated; stool culture; and abdominal ultrasound, as previously described [13–15].

### Adverse events

Physical examination was routinely performed. Pulmonary function assessment to evaluate possible bleomycin induced pulmonary toxicity (BIPT), including spirometry, was performed at baseline, interim evaluation, and at the end of treatment in all patients. Adverse events (AE) were classified as “bone-pain–related symptoms” with a comprehensive definition including the AE terms arthralgia, back pain, bone pain, neck pain, myalgia, and other musculoskeletal symptoms; skin evaluation data regarding possible erythematous reactions related to the *s.c.* G-CSF injection site was also retrieved. Data regarding possible gastrointestinal symptoms such as nausea, vomiting, and diarrhea were also reported. Safety was assessed by the incidence of all side effects related to the G-CSF schedule (either secondary and primary prophylaxis) and were reported according to the National Cancer Institute CTCAE v.5.0 [16].

### Statistical analysis

Patient and disease characteristics, FN rates, toxicity, and safety data were described as numbers and percentages or as median (range). Factors analyzed included age, B symptoms, large nodal masses (LNM), disease sites, Ann Arbor stage, and baseline risk group by prophylaxis. Continuous variables were summarized as mean ± SD or median (IQR) and compared using Student’s *t*-test or Wilcoxon rank test, as appropriate; categorical variables were compared using frequencies and percentages. A *P* < 0.05 was considered significant. Univariate logistic regression was performed for FN and for a composite endpoint (chemotherapy disruption or hospitalization); clinically relevant variables entered multivariate models regardless of univariate significance. Odds ratios (ORs) with 95% confidence intervals (CIs) were reported. Analyses were performed using SPSS version 26.0.

## Results

### Patient characteristics

**Fig. 1:**
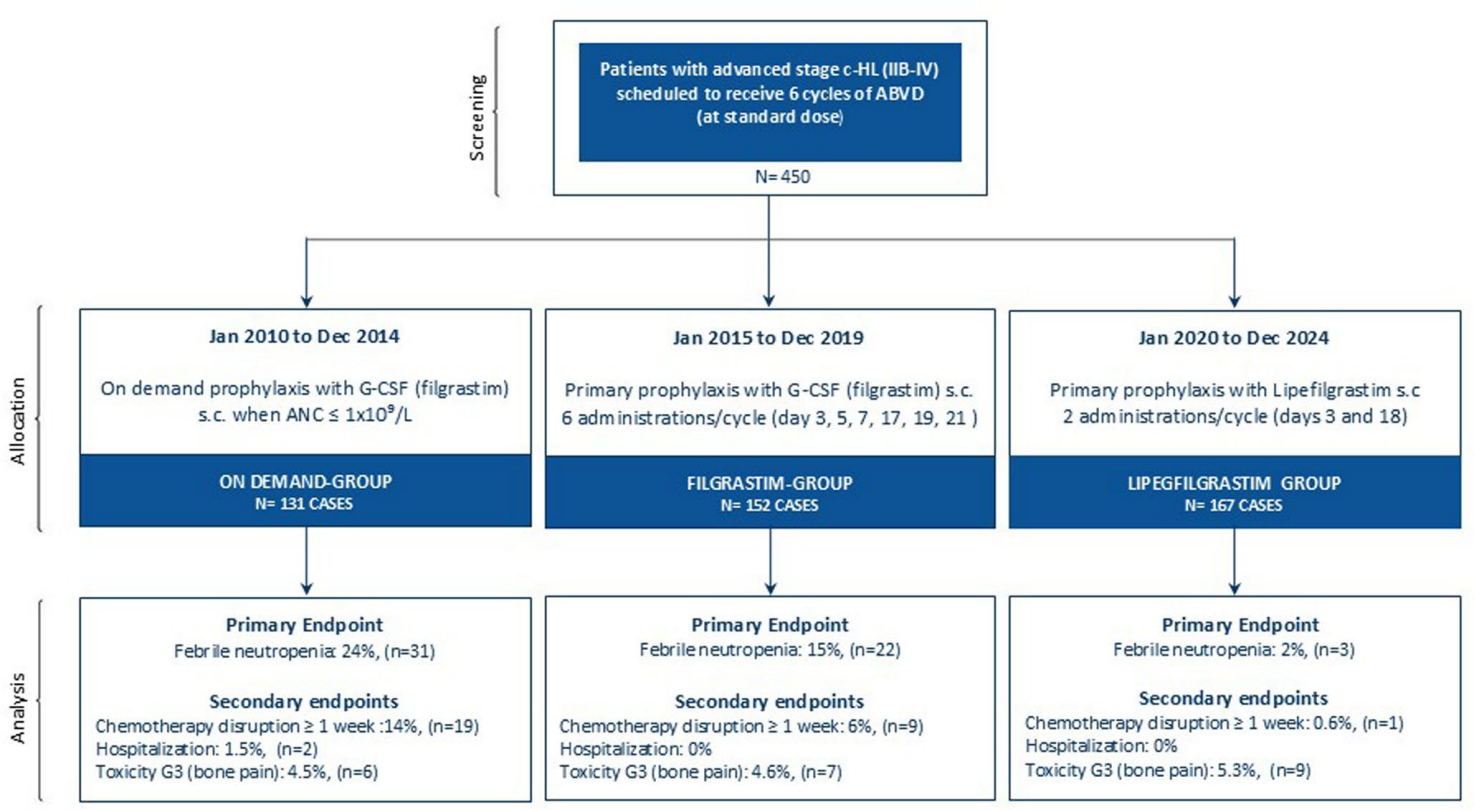
Flow chart of the patients throughout the study

From January 2010 to December 2024, 450 patients with untreated c-HL receiving front-line ABVD met the inclusion criteria and were enrolled in the final analysis. In particular, 131 patients from January 2010 to December 2014 were allocated in the *on-demand*-group receiving *s.c.* G-CSF secondary prophylaxis when ANC was ≤ 1 × 10^9^/L at the weekly CBC monitoring; 152 patients received filgrastim as primary prophylaxis from January 2015 to December 2019 with a 6-times administration (days 3, 5, 7, 17, 19 and 21) of each 4-week cycle of ABVD (*filgrastim*-group); and, from January 2020 to December 2024, 167 patients received primary prophylaxis with lipegfilgrastim *s.c.* as a single dose on days 3 and 18 of each 4-week cycle of ABVD (*lipegfilgrastim*-group). Table 1 shows the patients’ characteristics according to the different prophylaxis groups, and Fig. 1 shows the flowchart of patients’ allocation in the study. The median follow-up was 7 months (range, 6–8 months).

**Table 1:** Patients’ characteristics

	Total	On-demand group	Filgrastim group	Lipegfilgrastim group	*P*-value
	*n* (%)	*n* (%)	*n* (%)	*n* (%)	
**Number of patients**	450	131	152	167	0.112
**Male sex**	259 (57)	74 (56)	81 (53)	104 (62)	0.270
**Age**,** median (range)**	37.9 (18–88)	36.4 (18–80)	38.2 (18–86)	38.6 (19–88)	0.568
**Histological subtype**					
NS subtype	305 (68)	81 (62)	102 (67)	122 (73)	0.140
MC subtype	90 (20)	32 (24)	33 (22)	25 (15)	0.164
LR subtype	36 (8)	10 (8)	11 (7)	15 (9)	0.848
DL subtype	19 (4)	8 (6)	6 (4)	5 (3)	0.421
**B symptoms**	282 (63)	69 (53)	98 (64)	115 (69)	0.203
**Spleen involvement**	68 (15)	14 (11)	31 (20)	23 (14)	0.086
**Extra nodal involvement**	27 (6)	5 (4)	8 (5)	14 (8)	0.246
**Bulky disease**	119 (26)	30 (23)	47 (31)	42 (25)	0.392
**IPS risk factor**					
0–1	113 (25)	30 (23)	39 (26)	44 (26)	0.263
2–3	177 (39)	55 (42)	57 (37)	65 (39)	0.622
4–7	160 (36)	46 (35)	56 (37)	58 (35)	0.461
**Ann Arbor stage**					
II	181 (40)	58 (45)	54 (35)	69 (41)	0.395
III	137 (30)	33 (25)	53 (35)	51 (31)	0.981
IV	132 (30)	40 (30)	45 (30)	47 (28)	0.932

Values are n (%) unless otherwise specified

NS: nodular sclerosis; MC: mixed cellularity; LR: lymphocyte rich; LD: lymphocyte depleted

B symptoms: fever, weight loss > 10% in the last 6 months, nocturnal sweat. Bulky disease, defined as lymph node mass with long axis > 5 cm; Ann Arbor staging: stage I defined as one lymph node group; stage II defined as two lymph node groups on one side of the diaphragm; stage III, defined as multiple lymph node groups on both sides of the diaphragm; stage IV, defined as multiple extra-nodal sites or lymph nodes and extra-nodal disease

IPS: International Prognostic Score for advanced-stage Hodgkin lymphoma, it incorporates seven independent adverse prognostic factors: serum albumin < 4.0 g/dL, hemoglobin < 10.5 g/dL, male sex, age ≥ 45 years, Ann Arbor stage IV disease, leukocytosis (WBC ≥ 15,000/mm³), and lymphocytopenia (absolute lymphocyte count < 600/mm³ or < 8% of total WBC count)

Most patients were male (57%) and the median age across all patients was 37.9 years (range: 18–88). Regarding histological subtypes, the nodular sclerosis (NS) subtype was the most common observed in 305 patients (68%); the subtype distribution did not significantly differ among the groups. B symptoms were present in 282 patients (63%), while spleen involvement was reported in 68 patients (15%) and extranodal involvement in 27 patients (6%). None of these clinical features showed statistically significant differences between the treatment groups. Ann Arbor staging showed 181 patients (40%) in stage II (with local extra-nodal involvement [*n* = 15], or bulky disease [*n* = 70]), 137 (30%) in stage III, and 132 (30%) in stage IV, again with no significant variation between groups.

### Neutropenia episodes and G-CSF injections

Overall, a total of 85 neutropenia episodes were recorded: 52 (40%) in the *on-demand*-group, 30 (20%) in the *filgrastim*-group, and 3 (2%) in the *lipegfilgrastim*-group ([*P* < 0.001], Table 2). Grade 3 neutropenia occurred in 42 of the 52 episodes in the *on-demand*-group and in 25 of the 30 episodes in the *filgrastim*-group. Grade 4 neutropenia occurred in 10 episodes in the *on demand*-group and in 5 episodes in the *filgrastim*-group. In the *lipegfilgrastim*-group all episodes recorded were of grade 3. The median duration of neutropenia was 4 days (range, 2–8) in the *on demand-*group and 2 days (range, 2–4 and 1–3, respectively) in the *filgrastim-* and *lipegfilgrastim*-groups, respectively. The median time to neutrophil nadir was 7 weeks (range, 4–24) in the *on-demand*-group, 14 weeks (range, 10–20) in the *filgrastim*-group and 16 weeks (range, 14–21) in the *lipegfilgrastim*-group. The median number of G-CSF injections was: 25 (range 5–50) in the *on-demand*-group, 37 (range 32–44) in the *filgrastim*-group, and 12 (range 12–13) in the *lipegfilgrastim-*group.

**Table 2:** Characteristics of neutropenic episodes

Neutropenic episodes without infectious symptoms	On-demand group *N* = 131	Filgrastim Group *N* = 152	Lipegfilgrastim group *N* = 167
	52 (40)	30 (20)	3 (2)
Grade 3	42 (81)	25 (83)	3 (100)
Grade 4	10 (19)	5 (17)	0
Neutropenia duration, days, median (range)	4 (2–8)	2 (2–4)	2 (1–3)
Time to nadir, weeks, median (range)	7 (4–24)	14 (10–20)	16 (14–21)
Number of G-CSF injections, median (range)	25 (5–50)	37 (32–44)	12 (12–13)
**Febrile Neutropenia**	31 (24)	22 (14)	3 (2)
FUO	21 (16)	17 (11)	3 (2)
Clinically documented infections	8 (6)	4 (3)	-
*Respiratory tract infection*	4 (50)	3 (75)	-
*Gastrointestinal tract infection*	2 (25)	1 (25)	-
*Herpes Zoster infection*	2 (25)	0 (0)	-
Microbiologically documented infections*	2 (1.5)	1 (0.6)	-
*Staphylococcus spp.*	1 (50)	1 (100)	-
*Pseudomonas spp.*	1 (50)	-	-
**Chemotherapy disruptions due to FN**	19 (14)	9 (6)	1 (0.6)
Time delay	15 (11)	9 (6)	1 (0.6)
Dose disruption	4 (3)	-	-
Median delay, days (range)	10 (7–20)	8 (7–14)	7
**Hospitalization events due to FN**	2 (1.5)	-	-

FN: febrile neutropenia; G-CSF: granulocytes colony stimulating factor; FUO: fever of unknown origin. Values are n (%) unless otherwise specified. Each episode is recorded for single patient

*Regarding the microbiologically documented infection episodes: 2 patients had a bronchoalveolar lavage (BAL) positive for *Staphylococcus spp*. and 1 for *Pseudomonas spp*

### Primary endpoint

Overall, a total of 56 FN episodes were recorded during the chemotherapy period (Table 2). The incidence of FN was 24% (*n* = 31 FN episodes) for *on-demand*-group, 14% (*n* = 22) for *filgrastim*-group and 2% (*n* = 3) for *lipegfilgrastim*-group (*P* < 0.0001). In the *on-demand*-group of the 31 FN episodes eight resulted in a clinically documented infection: 4 patients had an infection of the respiratory tract (all with radiological signs of pneumonia) recorded at the 3rd cycle of ABVD for 2 patients and at the 5th cycle for other 2 patients; two had infection of the gastrointestinal tract (4th and 5th cycle of ABVD, respectively), the last 2 had cutaneous Herpes Zoster infection at the 5th and 6th cycle, respectively. Regarding the microbiologically documented infection episodes: 2 patients had a bronchoalveolar lavage (BAL) positive for *Staphylococcus spp*. and *Pseudomonas spp.*, respectively; the remaining 21 FN episodes were recorded as febrile of unknown origin (FUO).

In the *filgrastim*-group 4 of the 22 patients with FN had a clinically documented infection: 3 in the respiratory tract and 1 in the gastrointestinal tract, all episodes were recorded after the 5th cycle. For one patient a microbiologically documented infection was recorded: BAL positive for Staphylococcus spp.

No clinically or microbiologically documented infection was recorded in the *lipegfilgrastim-*group thus the 3 FN episodes were reported as FUO.

### Secondary endpoints

Chemotherapy disruption related to FN was observed in 14% of patients (*n* = 19) in the *on-demand-*group, with treatment delays (time disruption) occurring in 11% (*n* = 15) and dose reductions (dose disruption) in 3% (*n* = 4). Specifically, time disruptions were due to delays in treatment cycles, with a median delay of 10 days (range: 7–20 days). Dose disruptions involved a reduction in at least one drug, with dacarbazine alone reduced in three patients and both dacarbazine and doxorubicin in one patient. The median dose reduction was 85% of the planned dose (range: 75%–90%).

Two patients in the *on demand*-group were hospitalized due to FN, leading to chemotherapy disruption. The median hospitalization duration was 7 days (range: 2–10 days). Both patients were diagnosed with pneumonia (Table 2) and required intravenous antibiotic therapy and oxygen support. They were discharged with full recovery.

In the *filgrastim*-group, FN-related chemotherapy disruption was recorded in 6% of patients (*n* = 9), all of which were time disruptions with a median delay of 8 days (range: 7–14 days). No FN-related dose reductions or hospitalizations occurred in this group.

In the *lipegfilgrastim*-group, only one patient experienced chemotherapy disruption due to FN, consisting of a 7-day treatment delay (time disruption). No dose reductions or hospitalizations were recorded.

Grade ≥ 3 prophylaxis-related adverse events were recorded according to CTCAE v5.0 criteria. Overall, prophylaxis was well tolerated, with grade 3 toxicity (bone pain-related symptoms) occurring in only 5% of patients: 6 in the *on demand*-group, 7 in the *filgrastim*- group, and 9 in the *lipegfilgrastim*-group. These symptoms were successfully managed with paracetamol or tramadol (used in five patients with paracetamol-refractory pain).

In addition, no cases of clinically significant BIPT were observed in the overall cohort, regardless of the G-CSF prophylaxis strategy.

### Statistical analysis results

No significant statistical differences were found among the 3 groups of prophylaxis regarding the baseline characteristics (Table 1) while a significant difference was found for the number of neutropenia episodes (*P* < 0.001). The prophylaxis strategy was significantly associated with the endpoints of FN (*P* < 0.0001), chemotherapy disruption (*P* < 0.001), and hospitalization (*P* < 0.001).

In the univariate hazard analysis of the primary endpoint of FN, the protective effect was most pronounced with lipegfilgrastim, which was associated with a greater risk reduction compared with both filgrastim and *on-demand* prophylaxis (Table 3). Regarding the clinical characteristics, age < 50 years was significantly associated with a lower risk of FN (OR 0.29, 95% CI 0.11–0.77, *p* = 0.012). In the multivariate models, most clinical variables lost significance, and the prophylaxis strategy was still the only strong independent predictor (OR 0.19, 95% CI 0.11–0.35, *P* < 0.001).

**Table 3:** Univariate and multivariate analysis of the study endpoints

Variable	FN Univariate Cox proportional Hazard OR (95% CI), *P* value	FN Multivariate Cox Regression Analysis OR (95% CI), *P* value	CHT disruption/Hospitalization Univariate Cox proportional Hazard OR (95% CI), *P* value	CHT disruption/Hospitalization Multivariate Cox Regression Analysis OR (95% CI), *P* value
Age < 50 y	0.29 (0.11–0.77), 0.012	—	0.27 (0.05–1.36), 0.111	—
B symptoms	0.86 (0.39–1.91) 0.718	—	3.77 (1.10–12.93), 0.035	7.01 (0.94–52.2), 0.057
Bulky > 7 cm	1.30 (0.73–2.32), 0.380	—	0.91 (0.35–2.32), 0.840	—
Advanced Stage	1.40 (0.79–2.48), 0.248	—	1.30 (0.50–3.34), 0.591	3.02 (1.11–8.24), 0.031
Extranodal involvement	1.54 (0.56–4.21), 0.406	—	1.38 (0.51–3.71), 0.524	—
IPS ≥ 3	1.32 (0.66–2.62), 0.436	—	0.57 (0.13–2.41), 0.441	—
Prophylaxis (Peg-G-CSF/G-CSF/on demand)	χ² = 28.05, *p* < 0.0001†	0.19 (0.11–0.35), < 0.001	χ² = 42.53, *p* < 0.001†	NE§

† P-value from chi-squared test (univariate, logistic regression not estimable)

§ Model did not converge (complete separation)

The endpoints of chemotherapy disruption and hospitalization were analyzed as a composite endpoint, both in univariate and multivariate models, given the limited number of events and their overlap across individual outcomes; the univariate analysis showed that the presence of B symptoms was associated with a higher risk (OR 3.77, 95% CI 1.10–12.93, *P* = 0.035). In the multivariate model, advanced-stage emerged as an independent predictor (OR 3.02, 95% CI 1.11–8.24, *P* = 0.031), while B symptoms showed only a trend toward significance (OR 7.01, 95% CI 0.94–52.2, *p* = 0.057).

## Discussion

This multicenter retrospective study evaluated the impact of three G-CSF prophylaxis strategies on FN incidence in patients with high-risk c-HL treated with 6 courses of ABVD for advanced-stage disease. Our cohort represents a heterogeneous population of patients with advanced-stage c-HL, including individuals of all ages—from young adults to elderly patients—thus reflecting the real-world epidemiological profile of the disease in clinical practice. Although FN risk in ABVD is classified as low to intermediate by NCCN and ESMO guidelines [9], our real-world data spanning a period of 14 years, demonstrate a significantly higher incidence, particularly in the absence of structured prophylaxis. This discrepancy between guideline-based risk estimates and actual clinical outcomes highlights the limitations of risk stratification in daily practice. The rationale for adopting primary G-CSF prophylaxis in all patients treated with ABVD in our cohort should be interpreted in light of both real-world clinical experience and evolving guideline recommendations. While ABVD has historically been considered an intermediate-risk regimen for FN, our earlier cohorts consistently showed a clinically meaningful rate of neutropenic complications, supporting a preventive rather than reactive approach.

This consideration is especially relevant in adult patients, as reflected by the median age of approximately 40 years in our population. These findings are consistent with the conclusions of the recent Delphi consensus by Adamo et al. [17], which supports broader and individualized use of G-CSF, even for chemotherapy regimens traditionally categorized as intermediate risk, such as ABVD. As a matter of fact, the Delphi panel reached consensus that FN risk should be assessed at each chemotherapy cycle, and that long-acting G-CSF should be used 24–72 h post-chemotherapy, underscoring the importance of proactive prophylaxis in regimens like ABVD.

Our findings confirm the pivotal role of G-CSF prophylaxis in preventing febrile neutropenia, with lipegfilgrastim showing the most pronounced protective effect.

In our series, the *on-demand*-group, which received G-CSF only in response to ANC ≤ 1000/L, experienced the highest FN rate (24%), along with the greatest number of chemotherapy disruptions and hospitalizations, especially in patients older than 50 years and with advanced-stage and B symptoms. This suggests that reactive prophylaxis may be inadequate to ensure treatment continuity or prevent complications, particularly in high-risk patients. The *filgrastim*-group, receiving scheduled short-acting G-CSF, showed a reduced FN incidence (14%), with fewer treatment disruption and no hospitalizations. These results support a shift toward proactive management. However, the most favourable outcomes were observed in the *lipegfilgrastim*-group, where FN incidence dropped to 2%, with no hospital admissions or clinically documented infections. Moreover, fewer injections are required suggesting superior patient convenience.

All G-CSF formulations were well tolerated: grade ≥ 3 adverse events (per CTCAE v5.0), primarily bone pain, occurred in only 5% of cases and were manageable with standard analgesics such as paracetamol or tramadol.

In addition, in our study, no clinically significant BIPT was observed despite concomitant administration of bleomycin and G-CSF, likely reflecting the adoption of preventive strategies such as systematic bleomycin dose reduction in patients aged ≥ 60 years and regular pulmonary function monitoring; however, the relatively short follow-up precludes conclusions on late-onset toxicity.

The statistically significant reduction in FN rates observed across groups—from 24% in the *on-demand*-group to 14% with filgrastim and 2% with lipegfilgrastim (*P* < 0.0001)- was mirrored by a decrease in chemotherapy disruptions and hospitalizations, reinforcing the clinical benefits of a consistent prophylactic strategy.

A cost-effectiveness analysis was performed using official Italian price lists (AIFA, 2024), Diagnosis Related Groups, and the National Tariff Schedule. Considering both direct and indirect costs, including G-CSF acquisition, diagnostics, antibiotic therapy, and FN management, lipegfilgrastim showed a total cost of €209,348 per 100 patients, compared with €256,926 for on-demand G-CSF and €193,586 for filgrastim-based prophylaxis [18, 19]. Despite its higher acquisition cost (€192,000 for 12 doses at €160 each), lipegfilgrastim achieved a net saving of €47,578 versus the on-demand strategy, driven by fewer FN events and complications. The filgrastim group, while less expensive overall, had more clinically and microbiologically documented infections (four clinical—three respiratory, one gastrointestinal—and one microbiologically confirmed), requiring additional outpatient care and antibiotics. Conversely, the lipegfilgrastim arm reported no hospitalizations, no documented infections, and only three FN episodes (one managed in outpatient), minimizing resource use and preserving chemotherapy dose intensity. Thus, the long-acting G-CSF not only minimized healthcare utilization but also enhanced patient safety—supporting its role as a cost-effective option for FN prevention in c-HL treated with ABVD.

Although the frontline treatment landscape of advanced-stage classical Hodgkin lymphoma has evolved in recent years with the introduction of brentuximab vedotin plus AVD (A + AVD), ABVD remains widely used in real-world practice, and the demonstrated benefit of primary G-CSF prophylaxis is also clinically relevant in contemporary regimens such as A + AVD, where guideline recommendations support routine primary prophylaxis due to the higher risk of febrile neutropenia [2, 20].

Our study has several limitations: its retrospective design introduces the potential for selection bias. Additionally, the comparison across sequential time periods may reflect evolving standards of care unrelated to G-CSF use. Lastly, given the relatively short median follow-up of 7 months and the study design focused on treatment-related endpoints, we did not evaluate long-term outcomes such as PFS or OS; future studies should assess whether preserved dose intensity translates into improved survival.

In conclusion, our findings support the routine use of primary G-CSF prophylaxis in c-HL patients at high-risk receiving ABVD for six cycles, particularly with lipegfilgrastim. This strategy significantly reduces FN incidence and related complications, potentially enhancing the safety and effectiveness of curative chemotherapy.

## Data Availability

The data supporting the findings of this study are available within the article. Additional data is available at reasonable request to the corresponding author.
